# Cardiac monitoring and incidence of cardiotoxicity cardiomyopathy among breast cancer patients undergoing anthracycline regimen chemotherapy: insight from a single centre study

**DOI:** 10.1186/s12872-026-06470-9

**Published:** 2026-08-17

**Authors:** Astri Astuti, Adila Aafiyah, Aurora Adila Arderia, Melawati Hasan, Syarief Hidayat, Erwan Martanto, Mohammad R. Akbar

**Affiliations:** 1 Department of Cardiology and Vascular Medicine, Dr. Hasan Sadikin General Hospital, Bandung, West Java Indonesia; 2 Department of Cardiovascular Medicine, Faculty of Medicine, Universitas Padjadjaran, Bandung, Indonesia

**Keywords:** Anthracycline, Breast cancer, Cardiotoxicity, Chemotherapy

## Abstract

**Background:**

Anthracyclines remain a cornerstone of breast cancer therapy but carry a significant risk of cancer therapy-related cardiac dysfunction (CTRCD). This study evaluates the incidence of CTRCD in an Indonesian setting using the latest 2022 ESC Cardio-Oncology guidelines, focusing on subclinical markers such as high-sensitivity Troponin I (hs-cTnI), Global Longitudinal Strain (GLS) and Mechanical Dispersion (MD).

**Methods:**

This retrospective analytical cohort study involved 98 breast cancer patients treated with anthracyclines at a national referral hospital in Indonesia from July 2018 to February 2020. Clinical assessments, hs-cTnI, and echocardiography (LVEF, GLS, and MD) were performed at baseline, 1, 3, and 6 months. CTRCD was defined per the 2022 ESC criteria.

**Results:**

CTRCD occurred in 74.5% of patients, predominantly as asymptomatic mild cases (63.26%). While symptomatic CTRCD was relatively low (7.14%), asymptomatic dysfunction was detected as early as one-month post-chemotherapy. A significant progressive decline was observed in LVEF (68.2 ± 6.2% to 61.3 ± 8.8%, *p* < 0.001) and GLS (-19.7 ± 2.9% to -17.1 ± 3.5%, *p* < 0.001). Notably, mechanical dispersion significantly increased over time (*p* = 0.029), and median hs-cTnI surged from 1.6 ng/L to 82.2 ng/L (*p* < 0.001) by month 6.

**Conclusion:**

The high incidence of asymptomatic CTRCD underscores the inadequacy of relying on clinical symptoms alone. Integration of hs-cTnI, GLS, and mechanical dispersion monitoring is may be essential for early detection and enables timely cardioprotective intervention.

## Introduction

Breast cancer is one of the most common types of cancer in the world. In 2020, approximately 2.3 million women were diagnosed with breast cancer, and the mortality rate was 635,000 [1]. In Indonesia, breast cancer is the type of cancer with the highest number of patients and one of the leading causes of death. According to data from Global Cancer Statistics in 2020, the number of new cases of breast cancer in Indonesia reached 68,858, or 16.6% of the total 396,914 new cases, and the number of deaths reached more than 22,000 [2].

Chemotherapy is an important breakthrough in the treatment of cancer, but it is also associated with major adverse effects, especially cardiotoxicity [3]. The incidence rate of cancer therapeutic-related cardiac dysfunction (CTRCD) during or shortly after the completion of cancer therapy ranges from 9 to 26% after treatment with doxorubicin, 13–17% with trastuzumab, and 27–34% with combination therapy [4]. Anthracyclines are among the most commonly used chemotherapeutic drugs and have shown efficacy against several kinds of malignancies, particularly lymphoma and breast cancer. On the other hand, anthracyclines may cause progressive and irreversible cardiac damage [5, 6]. Cardiotoxicity due to anthracycline begins with damage to myocardial cells, followed by a decrease in left ventricular function, which, ultimately, if not treated, can cause heart failure [6]. Cardiotoxicity can be detected early and avoided by monitoring disease progression, including declines in left ventricular function, or by identifying subclinical myocardial injury through biomarker assessment, such as troponin measurement [7–9]. The recovery rate of cancer patients who are increasing in life expectancy afterwards also needs to be considered, and a focus on cardiac health has to be our priority. On the other hand, there is still much uncertainty in assessing cardiovascular risk in patients undergoing chemotherapy, especially in detecting subclinical changes [3]. There are several different definitions of cardiotoxicity, making it difficult to compare results across trials to determine the true incidence of various types of cardiovascular cardiotoxicity and CTRCD. Therefore, we aimed to assess the incidence of CTRCD in Indonesia, especially West Java, on the basis of the latest consensus definition of cardiovascular toxicity adopted in the 2022 European Society of Cardiology (ESC) cardio-oncology guidelines [10].

## Methods

This study was a retrospective analytical cohort study involving medical records of 98 breast cancer patients receiving chemotherapy with an anthracycline regimen at Dr. Hasan Sadikin General Hospital, Bandung. This research was conducted after obtaining ethical approval from the Research Ethics Committee (number 204/UN6). KEP/EC/2024 and research permission from the Director of Human Resources, Education and Research at Dr. Hasan Sadikin General Hospital, Bandung, with number DP.04.03/D.XIV.4.4/599/2024.

The inclusion criteria of this study included all breast cancer patients over the age of 18 years who were indicated to receive anthracycline regimens by an oncologist at Dr. Hasan Sadikin General Hospital, Bandung, from July 2018 to February 2020. The data that were assessed included clinical assessment and biomarker and echocardiography examination results at baseline and at 1, 3, and 6 months after the initiation of chemotherapy. The exclusion criterion in this study was patients with breast cancer whose medical records were illegible.

The definition of CTRCD was based on the ESC Cardio-Oncology Guideline 2022. Symptomatic CTRCD was defined as the presence of cardiac dysfunction with symptoms of heart failure resulting from cancer treatment and was grouped into very severe (heart failure requiring inotropic support, mechanical circulatory support, or consideration of transplantation), severe (heart failure hospitalization), moderate (requiring intensification of diuretic therapy and outpatient heart failure) and mild (mild symptoms of heart failure, no need for intensification of therapy). Asymptomatic CTRCD was defined as the absence of symptoms of heart failure resulting from cancer treatment. Asymptomatic CTRCD was grouped into severe (new decrease in Left ventricular Ejection Fraction (LVEF) to < 40%), moderate (new LVEF reduction by ≥ 10% points to an LVEF of 40–49% or new LVEF reduction by < 10% points to an LVEF of 40–49% accompanied by a decrease in Global Longitudinal Strain (GLS) of > 15% or a new increase in cardiac biomarkers) and mild (LVEF ≥ 50% and a decrease in GLS by > 15% from baseline and/or new increases in cardiac biomarkers) [10].

Global longitudinal strain (GLS, %) and mechanical dispersion (MD, Ms) were quantified via speckle-tracking echocardiography. Two-dimensional grayscale loops encompassing five cardiac cycles were acquired at a frame rate of 50–80 frames per second using General Electric (GE) Vivid 7 and T-8 systems. Offline analysis was performed using EchoPAC software (version 113) to evaluate GLS at end-systole immediately after aortic valve closure. The MD was defined as the standard deviation of the time-to-peak longitudinal strain across a 17-segment left ventricular model, measured from QRS onset [11]. 

High-sensitivity troponin I (hs-cTnI) was evaluated via the Vidas BioMérieux assay, which combines a one-step sandwich enzyme link fluorescent assay (ELFA). The global 99th percentile URL for high-sensitivity troponin I is 19 ng/L on the platforms used in this study.

Statistical analyses were performed using IBM SPSS Statistics. Continuous variables were tested for normality using the Shapiro-Wilk or Kolmogorov-Smirnov test. Normally distributed data are presented as mean ± standard deviation (SD), while non-normally distributed data are presented as median and interquartile range (IQR). Categorical variables are presented as frequency and percentage.

Baseline clinical characteristics, echocardiographic parameters, and hs-cTnI levels were compared between the normal and all CTRCD groups, and between the asymptomatic and symptomatic CTRCD groups. Continuous variables were analysed using the independent t-test for normally distributed data or the Mann-Whitney U test for non-normally distributed data. Categorical variables were analysed using the Chi-square test or Fisher’s exact test, as appropriate.

Serial changes in echocardiographic parameters and hs-cTnI levels of the patients who completed all follow-up time points from baseline to Month 1, Month 3, and Month 6 were analysed using repeated-measures ANOVA for normally distributed data or the Friedman test for non-normally distributed data. For repeated-measures ANOVA, post-hoc pairwise comparisons were performed using the Bonferroni correction. For the Friedman test, post-hoc pairwise comparisons were performed using the Wilcoxon signed-rank test with Bonferroni adjustment.

Multivariable logistic regression analysis using the Enter method was performed to identify factors independently associated with CTRCD. Results are reported as odds ratios (ORs), 95% confidence intervals (CIs), and p-values. Model performance was evaluated using the Omnibus test, Nagelkerke R², Hosmer-Lemeshow goodness-of-fit test, and overall classification accuracy.

CTRCD incidence was described as frequency and percentage at each follow-up time point. Incidence rates were calculated as the number of CTRCD events divided by total person-time at risk, expressed as events per 100 person-months, with 95% CIs.

## Results

**Fig. 1:**
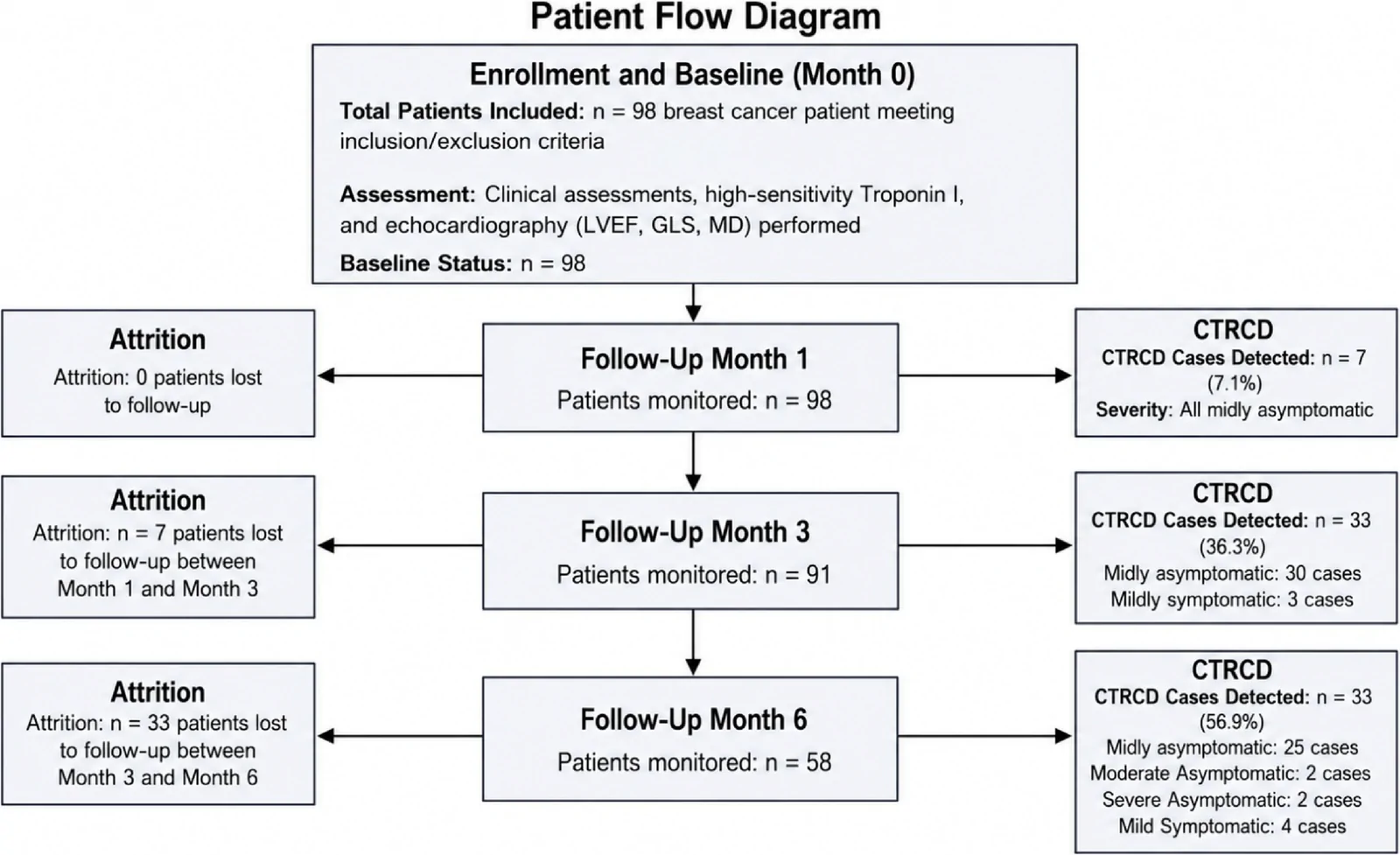
Patient flow diagram

Ninety-eight breast cancer patients underwent chemotherapy with an anthracycline regimen at Dr. Hasan Sadikin General Hospital, Bandung, who fulfilled the inclusion criteria. As illustrated in the Patient Flow Diagram in Fig. 1 and 98 breast cancer patients were enrolled at baseline (Month 0). At Month 1, all 98 patients were monitored, and 7 mildly asymptomatic CTRCD cases (7.1%) were detected. At Month 3, after 7 patients were lost to follow-up, 33 CTRCD cases (36.3%) were identified among the 91 remaining patients (30 mildly asymptomatic, 3 mildly symptomatic). By Month 6, an additional 33 patients were lost to follow-up, leaving 58 patients for evaluation. At this final assessment, 33 CTRCD cases (56.9%) were detected, comprising 25 mildly asymptomatic, 2 moderately asymptomatic, 2 severely asymptomatic, and 4 mildly symptomatic cases.

The average age of the patients was 47 ± 8 years, with no difference in asymptomatic and symptomatic CTRCD. Most of the patients (62.2%) were under 50 years old. The mean dose of anthracycline was 570 mg/m^2^ of body surface area, with no significant difference between the groups (*p* = 0.298). A total of 73 patients developed CTRCD (74.48%), 7 (7.14%) of whom were symptomatic, and the rest were asymptomatic.

A total of 41.8% of patients were classified as obese, and 30.6% were overweight based on body mass index (BMI) category. There was no significant variation in the distribution of BMI categories between the groups (*p* = 0.978). Among all patients, 20.4% had hypertension, with a greater prevalence in the asymptomatic CTRCD group (22.7%) than in the other groups; this difference was not statistically significant (*p* = 0.712). There was also no significant difference in diabetes mellitus between the groups (*p* = 0.161), with a higher rate of diabetes mellitus in patients with asymptomatic CTRCD (10.6%) than in those with normal and symptomatic CTRCD. Among all patients, 2% received mediastinal radiotherapy, with a greater proportion in the symptomatic CTRCD group (14.3%) than in the other groups.

Baseline characteristics did not differ significantly between patients who completed the 6-month protocol (* n=58*) and those who dropped out (* n=40*) (all * p>0.05*). No statistically significant differences were observed in mean age ($\:46.71\pm\:7.80$ vs. $\:47.63\pm\:7.92$ years, * p=0.570*), mean BMI ($\:25.71\pm\:3.78$ vs. $\:25.49\pm\:4.63{\text{ kg/m}}^{2}$, * p=0.798*), hypertension (25.9% vs. 12.5%, * p=0.107*), diabetes mellitus (6.9% vs. 7.5%, * p=1.000*), or cumulative anthracycline dose ($\:571.32\pm\:58.31$ vs. $\:554.5\pm\:86.84{\text{ mg/m}}^{2}$, * p=0.597*), confirming clinical homogeneity between the groups (Table 1).

**Table 1:** Baseline characteristics of breast cancer patients receiving chemotherapy and bivariate analysis with all CTCRD

Characteristic	Total (*n* = 98)	Total patients (*n* = 98)			Patients with CTRCD (*n* = 73)			Patients Follow-up Status (*n* = 98)		
		Normal (*n* = 25)	All CTRCD (*n* = 73)	*p* value	Asymptomatic CTRCD (*n* = 66)	Symptomatic CTRCD (*n* = 7)	*p* value	Complete (*n* = 58)	Dropout (*n* = 40)	*P* value
Age (years)	47 ± 8	49 ± 9	47 ± 7	0.263	47 ± 7	46 ± 9	0.533	46.71 ± 7.80	47.63 ± 7.92	0.570
Age Category (n, %)				0.788			0.855			0.640
< 50	61 (62.2)	15 (60.0)	46 (63.0)		42 (63.6)	4 (57.1)	0.911	35 (60.3)	26 (65.0)	
≥ 50	37 (37.8)	10 (40.0)	27 (37.0)		24 (36.4)	3 (42.9)		23 (39.7)	14 (35.0)	
BMI (kg/m^2^)	25.6 ± 4.1	25.7 ± 4.8	25.6 ± 3.9	0.864	25.6 ± 3.9	25.8 ± 4.4	0.974	25.71 ± 3.78	25.49 ± 4.63	0.798
BMI Category (n, %)				0.509			0.978			0.374
Underweight	2 (2.0)	1 (4.0)	1 (1.4)		1 (1.5)	0 (0.0)	0.880	0 (0.0)	2 (5.0)	
Normal	26 (26.6)	6 (24.0)	20 (27.4)		18 (27.3)	2 (28.6)		15 (25.9)	11 (27.5)	
Overweight	30 (30.6)	10 (40.0)	20 (27.4)		18 (27.3)	2 (28.6)		18 (31.0)	12 (30.0)	
Obesity	40 (41.8)	8 (32.0)	32 (43.8)		29 (43.9)	3 (42.9)		25 (43.1)	15 (37.5)	
Risk Factor (n, %)										
Hypertension	20 (20.4)	4 (16.0)	16 (21.9)	0.526	15 (22.7)	1 (14.3)	0.712	15 (25.9)	5 (12.5)	0.107
Diabetes Mellitus	7 (7.1)	0 (0.0)	7 (9.6)	0.186	7 (10.6)	0 (0.0)	0.161	4 (6.9)	3 (7.5)	1.000
Concomitant Diseases	1 (1.1)	0 (0.0)	1 (1.4)	1.000	1 (1.5)	0 (0.0)	0.783	1 (1.7)	0 (0.0)	1.000
History of Mediastinal Radiotherapy	2 (2.0)	0 (0.0)	2 (2.7)	1.000	1 (1.5)	1 (14.3)	0.053	2 (3.4)	0 (0.0)	0.512
Anthracycline Cumulative Dose (mg/m^2^), *n* = 51	570 ± 60	599 ± 100	567 ± 55	0.514	570 ± 52	540 ± 76	0.298	571.32 ± 58.31 (*n* = 47)	554.55 ± 86.84 (*n* = 4)	0.597

The comparison was performed between the normal and all CTRCD groups, the asymptomatic and symptomatic CTRCD groups, also the complete and drop out groups

A p-value < 0.05 was considered statistically significant

Continuous data are presented as mean ± SD and were analysed using the independent samples t-test. Categorical data are presented as n (%) and were analysed using the Independent t-test, Chi-square test or Fisher’s exact test, as appropriate

The mean LVEF values are shown in Table 2 and were similar across all groups, with a mean of 68.2% and minor variations between groups (67.2% in the normal group, 68.4% in the asymptomatic CTRCD group, and 69.5% in the symptomatic CTRCD group). There were no significant differences between the groups (*p* = 0.709).

**Table 2:** Baseline echocardiographic characteristics and troponin levels of breast cancer patients undergoing chemotherapy and bivariate analysis with all CTCRD

Variable	Total (*n* = 98)	Total patients (*n* = 98)			Patients with CTRCD (*n* = 73)		
		Normal (*n* = 25)	All CTRCD (*n* = 73)	*p* value	Asymptomatic CTRCD (*n* = 66)	Symptomatic CTRCD (*n* = 7)	*p* value
LVEF (%)	68.2 ± 6.2	67.2 ± 6.2	68.9 ± 6.7	0.291	68.4 ± 6.1	69.5 ± 9.3	0.709
GLS (%)	-19.7 ± 2.9	-18.4 ± 2.5	-20.1 ± 2.9	0.033*	-20.2 ± 2.9	-18.5 ± 1.7	0.051
LVEDV (ml)	83.7 ± 22.8	82.3 ± 23.6	79.0 ± 26.1	0.670	84.3 ± 23.3	81.5 ± 14.5	0.929
LVESV (ml)	24.0 (20.0–32.0)	25.0 (20.3–32.8)	24.0 (19.0–32.0)	0.585	24.0 (19.0–32.0)	20.5 (18.5–25.5)	0.540
LVSV (ml)	57.0 ± 15.6	54.9 ± 18.8	57.6 ± 14.7	0.505	57.6 ± 14.8	57.3 ± 14.8	0.801
TAPSE (mm)	21.3 (20.0–23.3)	21.3 (19.2–23.3)	21.3 (20.0–23.3)	0.952	21.1 (20.0–23.3)	22.5 (21.0–23.3)	0.706
E/A	1.09 ± 0.38	1.03 ± 0.39	1.11 ± 0.38	0.378	1.13 ± 0.38	0.87 ± 0.15	0.279
E/e’	8.65 ± 2.25	8.96 ± 2.32	8.56 ± 2.24	0.513	8.53 ± 2.26	9.24 ± 2.04	0.702
Mechanical Dispersion (Ms)	43.2 (36.5–48.0)	43.0 (34.4–56.6)	43.3 (37.4–47.1)	0.513	42.6 (36.3–46.7)	52.8 (46.6–54.9)	0.085
Hs-cTnI (ng/L)	1.6 (1.4–6.5)	1.4 (1.4–4.8)	2.1 (1.4–6.8)	0.309	2.7 (1.4–7.2)	1.8 (1.4–3.5)	0.345

*LVEF* Left Ventricular Ejection Fraction, *GLS* Global Longitudinal Strain, *LVEDV* Left Ventricular End-Diastolic Volume, *LVESV* Left Ventricular End Systolic Volume, *LVSV* Left Ventricular Stroke Volume, *TAPSE* Tricuspid Annular Plane Systolic Excursion

*A p-value < 0.05 was considered statistically significant

Normally distributed continuous variables are presented as mean ± SD and were analysed using the independent samples t-test. Non-normally distributed variables are presented as median (IQR) and were analysed using the Mann-Whitney U test. Comparisons were performed between the normal and all CTRCD groups, and between asymptomatic and symptomatic CTRCD groups

The global longitudinal strain (GLS) values ranged from − 18.4 ± 2.5% to -20.2 ± 2.9%, indicating decreased longitudinal function in the asymptomatic CTRCD group. However, this difference did not reach statistical significance (*p* = 0.051). The other echocardiographic parameters shown in Table 2 did not differ significantly between the groups. The median hs-cTnI level was normal at 1.6 (1.4–6.5) ng/L, with no difference between the groups (*p* = 0.345). Overall, these results indicate no significant differences in most baseline echocardiographic characteristics or troponin levels among the groups (Table 2).

Absolute baseline GLS was associated with CTRCD after adjusting for age, obesity, hypertension, baseline LVEF, and mechanical dispersion (OR = 1.297, 95% CI: 1.009–1.667, * p=0.042*). Other covariates, including age $\:\ge\:50$ years, obesity, hypertension, baseline LVEF, and mechanical dispersion demonstrated no significant associations with the outcome. However, the comprehensive multivariable model, stated in table 3, did not achieve overall statistical significance (Omnibus test * p=0.078*).

**Table 3:** Multivariable logistic regression analysis of factors associated with CTRCD

Variables	OR	95% CI	*p*-value
Age ≥ 50 years	0.421	0.102–1.728	0.230
Obesity	3.143	0.718–13.748	0.128
Hypertension	2.291	0.441–11.909	0.324
Absolute baseline GLS	1.297	1.009–1.667	0.042*
Hs-cTnI	0.998	0.994–1.003	0.451

Multivariable logistic regression analysis was performed using the Enter method. Results are presented as ORs, 95% CIs, and p-values. The dependent variable was CTRCD. Model performance was assessed using the Omnibus test, Nagelkerke R², Hosmer-Lemeshow goodness-of-fit test, and overall classification accuracy

Model Summary: The total number of analysed subjects was 73. The regression model was not statistically significant based on the Omnibus test (*p* = 0.078). The Nagelkerke R^2^ value was 0.199, indicating that the model explains approximately 19.9% of the variance in CTRCD occurrence. The Hosmer-Lemeshow test demonstrated a good fit for the data (*p* = 0.740). The overall classification accuracy of the model was 79.5%

*A p-value < 0.05 was considered statistically significant

**Fig. 2:**
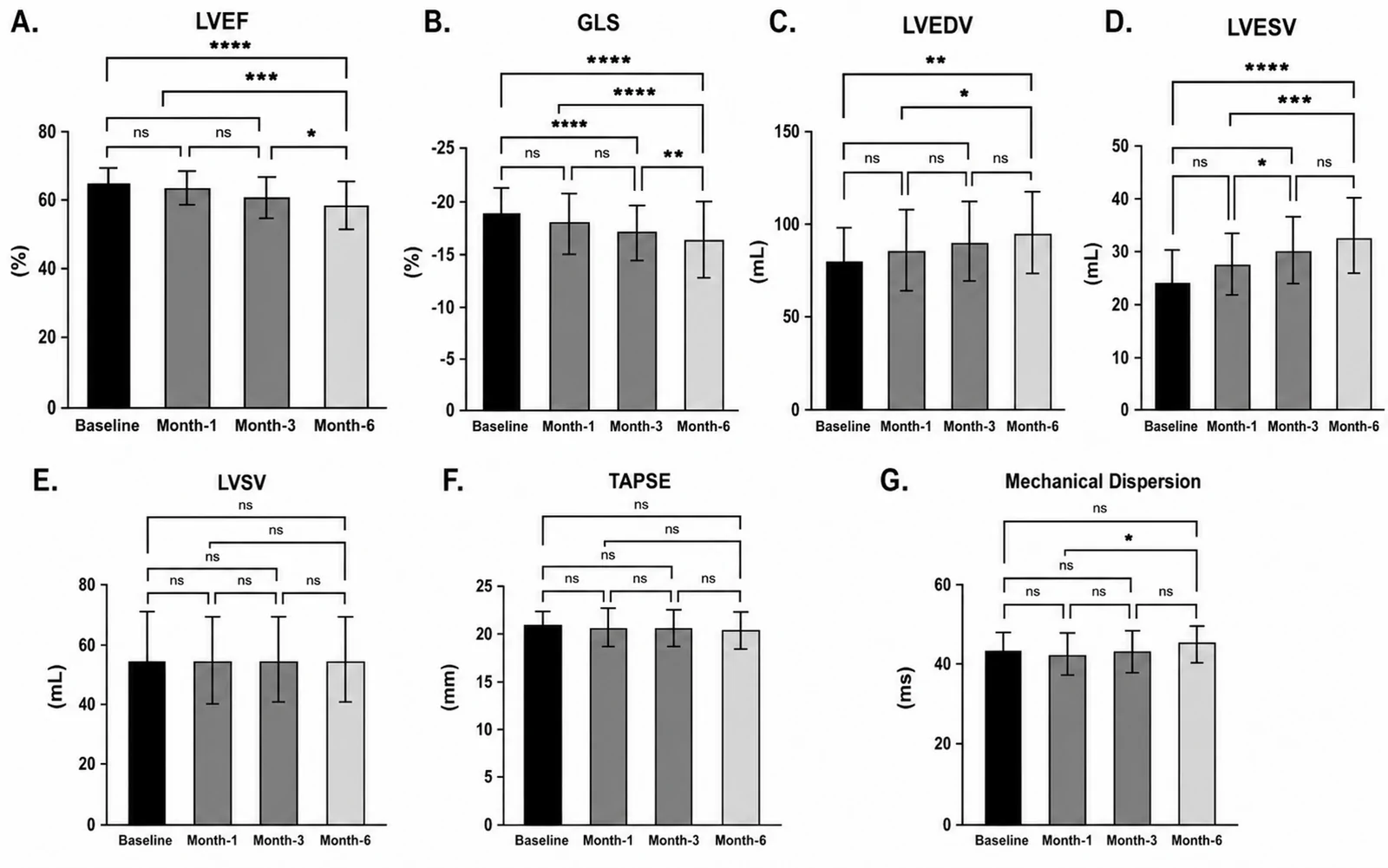
Changes in the mean echocardiographic characteristics of breast cancer patients receiving chemotherapy at baseline, month 1, month 3, and month 6 of follow-up. **A** There were significant LVEF changes from baseline to month 3, from baseline to month 6, from month 1 to month 6, and from month 3 to month 6. **B** GLS decreased significantly from baseline to month 3 and from month 6 onward, while no significant differences were observed in the first month. **C** The LVEDV increased significantly from baseline to month 3 and month 6, with no significant differences among the postbaseline measurements. **D** The LVESV was significantly greater at month 3 and month 6 than at baseline and from month 1 to month 6. **E** No significant differences in the LVSV were observed across the time points. **F** No significant differences in TAPSE were observed across time points. **G** A significant increase in mechanical dispersion was observed from month 1 to month 6. Notes: Serial changes in echocardiographic parameters were analysed using repeated-measures ANOVA for normally distributed variables and the Friedman test for non-normally distributed variables. Post-hoc pairwise comparisons were performed using Bonferroni correction for repeated-measures ANOVA and Wilcoxon signed-rank test with Bonferroni adjustment for Friedman test. A p-value < 0.05 was considered statistically significant

At 6 months of follow-up, a significant decrease was observed in LVEF among all patients who underwent chemotherapy, as stated in Table 4; Fig. 2, from 68.2% at baseline to 61.3% (*p* < 0.001), and in GLS values, from − 19.7 at baseline to − 17.1 at the 6th month (*p* < 0.001). The decrease became significant at the third month of follow-up compared with baseline, and it was even more pronounced at month 6. This finding indicates a progressive decline during therapy, especially after 3 months of follow-up. The left ventricular end-diastolic volume (LVEDV) and left ventricular end-systolic volume (LVESV) increased significantly after the baseline measurement and remained elevated throughout the 6-month follow-up, with a significant increase starting from the third month of follow-up (LVEDV: 83.7–90.0 mL; LVESV: 24.0–31.0 mL, *p* < 0.001). However, there were no significant differences between month-1, month-3, and month-6. This may indicate progressive cardiac remodelling along with a reduction in systolic function during chemotherapy.

**Table 4:** Echocardiographic characteristics of breast cancer patients receiving chemotherapy

Variable	Baseline	Month-1	Month-3	Month-6	*p* value
LVEF	68.2 ± 6.2	66.3 ± 6.6	64.5 ± 6.7	61.3 ± 8.8	< 0.001*
GLS	-19.7 ± 2.9	-19.2 ± 2.7	-18.6 ± 2.3	-17.1 ± 3.5	< 0.001*
LVEDV	83.7 ± 22.8	86.2 ± 21.8	90.0 ± 21.9	94.4 ± 21.2	< 0.001*
LVESV	24.0 (20.0–32.0)	27.0 (20.8–32.3)	31.0 (25.0–38.0)	34.0 (27.0–44.0)	< 0.001*
LVSV	57.0 ± 15.6	58.4 ± 16.4	58.0 ± 16.3	56.9 ± 12.5	0.759
TAPSE	21.3 (20.0–23.3)	21.3 (19.1–23.8)	21.1 (19.0–23.0)	21.0 (20.0–22.2)	0.729
E/A	1.09 ± 0.38	1.11 ± 0.61	1.07 ± 0.38	1.15 ± 0.49	0.345
E/e’	8.65 ± 2.25	8.24 ± 2.44	8.47 ± 2.39	9.49 ± 3.49	0.020*
Mechanical Dispersion	43.2 (36.5–48.0)	42.3 (36.3–47.7)	43.6 (38.9–50.4)	46.3 (41.7–51.7)	0.029*

Serial changes in echocardiographic parameters from baseline to Month 1, Month 3, and Month 6 were analysed using repeated-measures ANOVA for normally distributed variables and the Friedman test for non-normally distributed variables. Post-hoc pairwise comparisons were performed using Bonferroni correction for repeated-measures ANOVA and Wilcoxon signed-rank test with Bonferroni adjustment for the Friedman test

*LVEF* Left Ventricular Ejection Fraction, *GLS* Global Longitudinal Strain, *LVEDV* Left Ventricular End-Diastolic Volume, *LVESV* Left Ventricular End-Systolic Volume, *LVSV* Left Ventricular Stroke Volume, *TAPSE* Tricuspid Annular Plane Systolic Excursion

*A p-value < 0.05 was considered statistically significant.

In terms of the echocardiography parameters, the E/e’ ratio significantly increased at the 6th month of follow-up (*p* = 0.020), indicating an increase in left ventricular diastolic filling pressure. However, the E/A ratio did not significantly change (*p* = 0.345). On the other hand, the mechanical dispersion value significantly increased over time (*p* = 0.029).

Overall, these findings demonstrate that chemotherapy is associated with a progressive decline in systolic function, accompanied by structural remodelling, altered diastolic filling pressures, and increased mechanical dispersion, which may contribute to subclinical cardiotoxicity within six months.

**Fig. 3:**
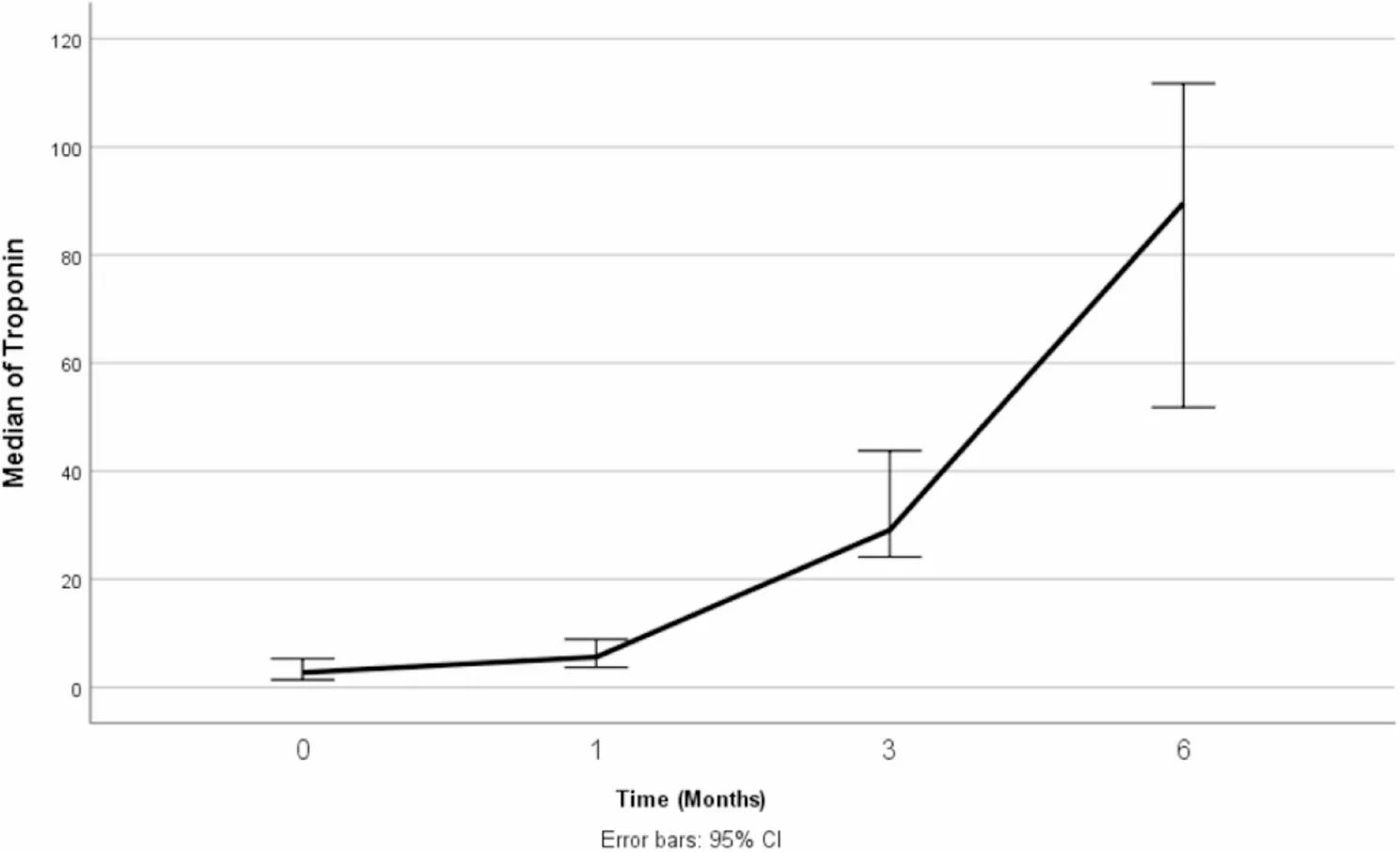
Hs-cTnI changes at months 1, 3, and 6 during chemotherapy. Notes: Hs-Troponin I levels are presented as median values over time. Serial changes were analysed using the Friedman test because troponin data were non-normally distributed. Post-hoc pairwise comparisons were performed using the Wilcoxon signed-rank test with Bonferroni adjustment. A p-value < 0.05 was considered statistically significant

Hs-cTnI levels (Table 5) increased significantly and progressively, from a median of 1.6 ng/L at baseline to 82.2 ng/L at six months of follow-up (*p* < 0.001). By the third month (Fig. 3), the median score was already above the 99th percentile, suggesting that a significant proportion of patients experienced cardiac injury or stress after chemotherapy. During the follow-up, 62 patients (63%) had hs-cTnI values > 19 ng/L.

**Table 5:** Troponin levels in patients with breast cancer receiving chemotherapy

Variable	Baseline	Month-1	Month-3	Month-6	*p* value
Hs-cTnI	1.6 (1.4–6.5)	4.6 (1.6–11.2)	29.7 (20.0-60.9)	82.2 (38.5-151.2)	< 0.001*

Hs-cTnI levels are presented as median (IQR). Serial changes from baseline at Month 1, Month 3, and Month 6 were analysed using the Friedman test, followed by Wilcoxon signed-rank post-hoc tests with Bonferroni adjustment when significant

*A p-value < 0.05 was considered statistically significant

As shown in Tables 6 and 7 cases of CTRCD were found in the first month of the 6-month follow-up period (7.14% of all patients), all of which were mildly asymptomatic. This number increased significantly to 33 of 91 undermonitored patients (36.26%) at the third month, with 30 patients categorised as mildly asymptomatic (32.97%) and 3 as mildly symptomatic (3.30%). Among the 7 mild symptomatic patients, 1 high-risk patient had cardiotoxicity due to a history of radiotherapy in the mediastinum and obesity, and 1 moderate-risk patient had a history of hypertension and obesity based on the HFA-ICOS (Heart Failure Association – International Cardio-Oncology Society) score. Four symptomatic patients were initially diagnosed as asymptomatic CTRCD at month 3.

**Table 6:** Number of CTRCD cases among breast cancer patients receiving chemotherapy

Characteristics		Baseline		Month-1		Month-3		Month-6
	*n*	%	*n*	%	*n*	%	*n*	%
All CTRCD	0	0%	7/98	7.1%	33/91	36.3%	33/58	56.9%
Mild asymptomatic	0	0%	7/98	7.1%	30/91	32.9%	25/58	43.1%
Moderate asymptomatic	0	0%	0	0%	0	0%	2/58	3.4%
Severe asymptomatic	0	0%	0	0%	0	0%	2/58	3.4%
Mild symptomatic	0	0%	0	0%	3/91	3.3%	4/58	6.9%

Data are presented as frequency and percentage. CTRCD cases were described at each follow-up time point according to the 2022 ESC Cardio-Oncology Guidelines and classified into mild asymptomatic, moderate asymptomatic, severe asymptomatic, and mild symptomatic CTRCD

*CTRCD* Cancer Therapy-Related Cardiac Dysfunction

**Table 7:** Incidence Rate of Cardiotoxicity Cardiomyopathy in Breast Cancer Patients Undergoing Chemotherapy

Outcome	Total	Event	Time at Risk	Rate (95% CI)	Rate in 100 person-month
All CTRD	98	73	454	0.161 (0.128–0.202)	16.1 (12.8–20.2)
Symptomatic CTRCD	98	7	454	0.015 (0.007–0.032)	1.5 (0.7–3.2)
Asymptomatic CTRCD	98	66	454	0.145 (0.114–0.185)	14.5 (11.4–18.5)

Incidence rates were calculated by dividing the number of CTRCD events by total person-time at risk and expressed as events per person-month and per 100 person-months. The 95% confidence intervals were calculated for each incidence rate. Separate incidence rates were calculated for all CTRCD, symptomatic CTRCD, and asymptomatic CTRCD

The number of cases continued to increase until the sixth month, when more than half of the undermonitored patients (33 of 58; 56.9%) experienced CTRCD. Four patients (6.9%) experienced mild symptomatic, 25 patients (43.1%) experienced mild asymptomatic, 2 patients experienced moderate asymptomatic (3.4%), and 2 patients experienced severe asymptomatic CTRCD (3.4%). Although the number of symptomatic patients has increased, its incidence remains lower than that of asymptomatic CTRCD patients, indicating that most patients are asymptomatic.

The overall incidence rate of CTRCD was calculated by accounting for the actual observed time at risk for each individual patient until the event occurred, loss to follow-up, or study completion. Among the 98 patients tracked over a total accumulated individual risk period of 454 person-months, 73 CTRCD events occurred. This corresponds to an overall incidence density rate of 16.1 events per 100 person-months (95% CI: 12.8–20.2). Specifically, the incidence rate for asymptomatic CTRCD was 14.5 per 100 person-months (95% CI: 11.4–18.5), whereas for symptomatic CTRCD, it was significantly lower at 1.5 per 100 person-months (95% CI: 0.7–3.2), as detailed in Table 7.

**Fig. 4:**
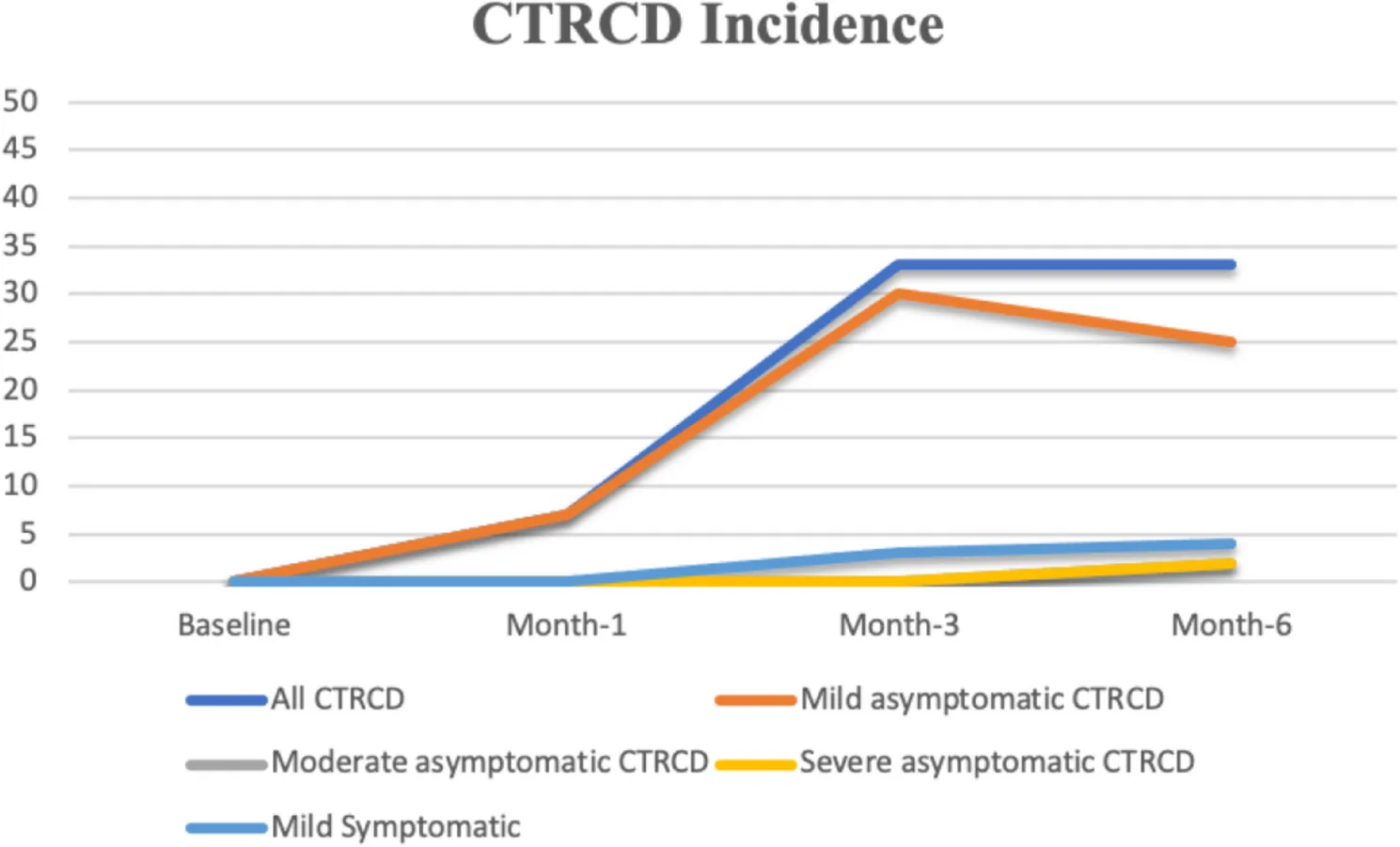
CTRCD incidence in breast cancer patients receiving chemotherapy at 1, 3, and 6 months. Notes: CTRCD incidence is presented descriptively as frequency and percentage at each follow-up time point

Figure 4 shows that the incidence of CTRCD increased significantly during therapy, peaking at month 3. Most cases are mild and asymptomatic, whereas severe or symptomatic cases are less common (Table 8).

**Table 8:** Exploratory sensitivity analysis of CTRCD incidence according to baseline hypertension and obesity status

Baseline risk factor	Normal	CTRCD	*p*-value
Hypertension status			0.526
Without hypertension	21/78 (26.9%)	57/78 (73.1%)	
With hypertension	4/20 (20.0%)	16/20 (80.0%)	
Obesity status			0.299
Non-obese	17/58 (29.3%)	41/58 (70.7%)	
Obese	8/40 (20.0%)	32/40 (80.0%)	

Data are presented as n/N (%). CTRCD incidence was compared between patients with and without baseline hypertension, and between obese and non-obese patients. The Chi-square test was used for group comparisons. A p-value < 0.05 was considered statistically significant

To explore the influence of baseline cardiovascular risks, a stratified sensitivity analysis was conducted to evaluate the impact of baseline hypertension and obesity on the occurence of cardiotoxicity (Table 8). The incidence of CTRCD was 80.0% (16 out of 20) in patients with baseline hypertension, compared to 73.1% (57 out of 78) in those without hypertension. This incremental variance in incidence density between the two subgroups was not statistically significant (*p* = 0.526), suggesting a widespread toxic impact of anthracyclines regardless of baseline blood pressure status.

In addition, the incidence of CTRCD was 80.0% (32 out of 40) among patients with baseline obesity, compared with 70.7% (41 out of 58) in the non-obese subgroup. Although the absolute rate of cardiotoxicity was higher in obese individuals, this variance did not reach statistical significance (*p* = 0.509), indicating that the anthracycline regimen posed a substantial cardiotoxic risk across all body mass index categories in this cohort.

## Discussion

### Principal findings and guideline comparisons

This study demonstrates a 74.5% cumulative incidence of cancer therapy-related cardiac dysfunction (CTRCD) among breast cancer patients undergoing anthracycline chemotherapy over a 6-month follow-up, calculated based on the actual observed time-at-risk. Notably, 67.34% of the cohort remained asymptomatic, with the vast majority classified as mild asymptomatic CTRCD (63.26%), while only 7.14% developed symptomatic manifestations. The corresponding overall incidence rate was 16.1 events per 100 person-months (95% CI: 12.8–20.2), heavily driven by the asymptomatic risk tier at 14.5 events per 100 person-months.

This high incidence is primarily driven by adherence to the contemporary 2022 ESC Cardio-Oncology guidelines [10]. This modern framework recognises subclinical markers—specifically a relative global longitudinal strain (GLS) reduction of $\:>15\%$ and/or new elevations in high-sensitivity cardiac troponin I (hs-cTnI) above the 99th percentile URL—as definitive criteria for mild asymptomatic CTRCD, even in the setting of a preserved LVEF ($\:\ge\:50\%$) [10, 11]. Consequently, capturing these early-stage subclinical cellular disruptions expands the diagnostic threshold compared to historical datasets. Older criteria, such as the 2016 ESC position paper [9], relied heavily on macro-structural changes (an LVEF reduction of $\:\ge\:10$ percentage points to a subnormal value) or overt clinical symptoms, thereby masking early-stage cardiac dysfunction and yielding lower reported cardiotoxicity rates ranging from 9% to 26% [4, 6].

Our findings align conceptually with recent trials tracking subclinical myocardial injury. The CARDIOTOX registry identified cardiotoxicity in 37.5% of patients during follow-up, with mild cases predominating (31.6%) [12]. Similarly, applying the 2022 ESC consensus definitions, Oristrell et al. reported an asymptomatic CTRCD rate of 60%, with 55.8% classified as mild [9, 13]. Mecinaj et al. also noted that 47.8% of patients presented with mild asymptomatic CTRCD post-anthracycline therapy, driven almost entirely by early biomarker elevations [14].

### Temporal trends and ventricular remodelling

Temporally, subclinical injury emerged as early as month 1 (7.1%) and rose progressively by month 3 (36.3%) and month 6 (56.9%). This deterioration is objectively demonstrated by our serial longitudinal metrics. Over 6 months, significant progressive declines occurred in mean LVEF ($\:68.2\pm\:6.2\%$ to $\:61.3\pm\:8.8\%$, * p<0.001*) and absolute GLS magnitude ($\:-19.7\pm\:2.9\%$ to $\:-17.1\pm\:3.5\%$, * p<0.001*), with changes becoming markedly more pronounced after the third month. These functional impairments were accompanied by adverse structural remodelling, as evidenced by significant progressive increases in both LVEDV and LVESV (* p<0.001*). Furthermore, a significant longitudinal rise in the E/e’ ratio (* p=0.020*) and mechanical dispersion (* p=0.029*) indicates elevated left ventricular diastolic filling pressures and contraction heterogeneity, reflecting active myocardial stress induced by progressive anthracycline exposure [15–17].

This active subclinical damage is further corroborated by our biomarker kinetics, in which the median hs-cTnI increased from 1.6 ng/L at baseline to 82.2 ng/L by month 6 (*p* < 6 (* p<0.001*). While older literature traditionally linked troponin release to chronic systolic failure [7, 8], modern high-sensitivity assays successfully capture a broader spectrum of transient cellular stress [18, 19]. However, clinical caution is warranted when interpreting isolated subclinical findings. As demonstrated by Mecinaj et al., transient troponin elevations during adjuvant chemotherapy are not inevitably followed by permanent systolic dysfunction or hard clinical events at extended 24-month follow-up [14, 20].

### Multivariable and stratified sensitivity analyses

An exploratory multivariable logistic regression model indicated that absolute baseline GLS was associated with CTRCD (OR = 1.297, 95% CI: 1.009–1.667, * p=0.042*). However, the overall model did not achieve statistical significance (Omnibus test * p=0.078*), accounting for only a modest fraction of the outcome variance (Nagelkerke $\:{R}^{2}=0.199$). This mathematical constraint is likely an artefact of our single-centre sample size, a narrow baseline strain distribution, and an uneven distribution of outcomes. Furthermore, because the diagnostic definition of CTRCD inherently incorporates a relative longitudinal decline from a patient’s own baseline strain, the non-significant Omnibus test and wide confidence intervals necessitate interpreting this association strictly as an exploratory, hypothesis-generating trend rather than a confirmed independent predictor, and warrant verification in larger, adequately powered multi-centre cohorts. 

To minimise interpretive bias across conventional risk factors under these statistical constraints, univariable stratified sensitivity analyses were conducted. CTRCD incidence did not differ significantly between patients with and without baseline hypertension (80.0% vs. 73.1%, *p* = 0.526) or between obese and non-obese individuals (80.0% vs. 70.7%, *p* = 70.7%, *p* = 0.299). A plausible explanation is that the uniform, high-dose exposure to anthracycline-based chemotherapy (570 ± 60mg/m^2^) and the exceptionally high overall incidence of mild asymptomatic CTRCD blunted our statistical power to detect differences across conventional risk-factor strata [10].

### Study limitations and generalizability

The retrospective analytical design of this study and the substantial longitudinal patient attrition introduce important limitations, particularly the risk of selection bias. As illustrated in the Participant Flow Diagram (Fig. 4), the monitored cohort decreased from 98 patients at baseline to 91 at month 3, and further declined to 58 by month 6. This attrition was primarily driven by real-world logistical barriers, such as out-of-town patients opting to continue subsequent chemotherapy cycles or routine check-ups at regional hospitals closer to their hometowns, or missing elective screening appointments.

This selective retention introduces a potential dual-directional bias, risking either overestimating the cumulative incidence at later stages if higher-risk or subtly symptomatic patients were more compliant with surveillance imaging, or underestimating the cumulative incidence at later stages if patients lost to follow-up developed undetected or unrecorded CTRCD. However, a comparative analysis between participants who completed the 6-month follow-up (“completers,” n = 58) and those who did not (“non-completers,” n = 40) revealed no statistically significant differences in baseline demographic characteristics, clinical comorbidities, LVEF, or GLS (all p > 0.05). This similarity indicates that participants who were lost to follow-up were not systematically different from those who remained in the study with respect to the measured baseline variables. Therefore, the risk of substantial attrition bias is reduced, and the reported 6-month incidence rates are less likely to have been materially influenced by baseline differences between completers and non-completers. Although these findings are consistent with an ‘Missing Completely at Random’ (MCAR) or ‘Missing at Random’ (MAR) mechanism, they do not definitively prove the underlying mechanism of missingness.

Nevertheless, this attrition limited our capacity to evaluate hard clinical endpoints, such as overall mortality or acute decompensated heart failure episodes, which are vital for mapping the long-term clinical trajectory of mild asymptomatic CTRCD. Additional diagnostic limitations include the lack of a formalised baseline risk classification model (e.g., HFA-ICOS scoring) and the absence of baseline or serial NT-proBNP assessments, which restricted our ability to evaluate early hemodynamic wall stress.

Furthermore, the generalizability of our findings requires cautious interpretation. Conducted at a single national referral centre, this cohort reflects a specific Indonesian demographic characterised by a younger age distribution (62.2% * <50* years) and a high prevalence of baseline obesity (41.8%). Given distinct regional disparities in healthcare infrastructure and local capacity to monitor advanced metrics (such as GLS and hs-cTnI), our 74.5% incidence primarily serves as a regional benchmark within the Indonesian healthcare framework. It underscores the urgent need for collaborative, prospective multi-centre registries to accurately map trans-regional cardiotoxic trends across diverse Indonesian or South East Asian populations.

### Practical clinical implications

From a practical clinical perspective, our results provide definitive evidence supporting a shift toward proactive cardiac surveillance, aligning with regional calls to enhance cardio-oncology awareness and early surveillance in Asian populations [21]. Relying solely on clinical symptoms is inadequate, as the vast majority of CTRCD events in this cohort were entirely mild and asymptomatic (63.3%). Our data demonstrate that identifying subclinical enzyme leaks and subtle strain drops provides a vital therapeutic window. According to the 2022 ESC Cardio-Oncology guidelines, cardioprotective therapy with ACEIs, ARBs, and/or beta-blockers may be considered for mild asymptomatic CTRCD (Class IIa, Level of Evidence B) to arrest further functional decline [10]. However, the initiation of these neurohormonal antagonists must be balanced carefully against potential treatment-related side effects—including systemic hypotension, electrolyte imbalances, and progressive worsening of renal function—particularly in cancer patients undergoing intensive oncology treatments.

## Conclusion

Anthracycline-induced cardiotoxicity manifests dynamically, with mild asymptomatic CTRCD emerging as early as one month post-chemotherapy and substantially outnumbering symptomatic cases, which typically surface by the third month. Despite the absence of overt clinical symptoms, these patients can experience progressive subclinical myocardial changes, characterised by left ventricular remodelling, elevated diastolic filling pressures, and increased heterogeneity of contraction. These findings support the implementation of proactive cardio-oncology surveillance frameworks that incorporate serial, multimodal tracking with GLS and high-sensitivity biomarkers. Acknowledging the constraints of a single-centre retrospective cohort design, our recommendations should be interpreted as institutional insights rather than generalised clinical mandates. Nevertheless, this study underscores that early subclinical detection offers a critical monitoring period to help guide timely clinical decisions and optimise the cardiac care of breast cancer survivors.

## Data Availability

The authors commit to sharing all manuscript materials and underlying raw data with the scientific community for non-commercial research, provided such requests maintain the strict anonymity and confidentiality of study participants.

## Abbreviations

BMI: Body mass index
CTRCD: Cancer therapy-related cardiac dysfunction
ESC: European Society of Cardiology
ELFA: Enzyme Link fluorescent Assay
GLS: Global Longitudinal Strain
HFA-ICOS: Heart Failure Association – International Cardio-Oncology Society
Hs-cTnI: High-sensitivity cardiac troponin I
LVEDV: Left Ventricular End-Diastolic Volume
LVEF: Left ventricular Ejection Fraction
LVESV: Left Ventricular End-Systolic Volume
LVSV: Left Ventricular Stroke Volume
MD: Mechanical dispersion
ROS: Reactive oxygen species
TAPSE: Tricuspid Annular Plane Systolic Excursion
URL: Upper Reference Limit
